# The Immune Microenvironment in Mesothelioma: Mechanisms of Resistance to Immunotherapy

**DOI:** 10.3389/fonc.2019.01366

**Published:** 2019-12-06

**Authors:** Gerard J. Chu, Nico van Zandwijk, John E. J. Rasko

**Affiliations:** ^1^Gene and Stem Cell Therapy Program Centenary Institute, University of Sydney, Department of Immunology, Royal Prince Alfred Hospital, Sydney, NSW, Australia; ^2^Sydney Medical School, Sydney Local Health District (Concord Repatriation General Hospital), University of Sydney, Sydney, NSW, Australia; ^3^Gene and Stem Cell Therapy Program Centenary Institute, University of Sydney, Cell & Molecular Therapies, Royal Prince Alfred Hospital, Sydney, Sydney Medical School, University of Sydney, Sydney, NSW, Australia

**Keywords:** mesothelioma, microenvironment, immunotherapy, tumor-associated macrophages, myeloid-derived suppressor cells, T-cells

## Abstract

Although mesothelioma is the consequence of a protracted immune response to asbestos fibers and characterized by a clear immune infiltrate, novel immunotherapy approaches show less convincing results as compared to those seen in melanoma and non-small cell lung cancer. The immune suppressive microenvironment in mesothelioma is likely contributing to this therapy resistance. Therefore, it is important to explore the characteristics of the tumor microenvironment for explanations for this recalcitrant behavior. This review describes the stromal, cytokine, metabolic, and cellular milieu of mesothelioma, and attempts to make connection with the outcome of immunotherapy trials.

## Introduction

Malignant pleural mesothelioma (MPM) has a justified reputation for being resistant to therapy. Large case series of patients with mesothelioma indicate a median overall survival of only 9.5 months (1). The epithelioid histological subtype is the most common variant; it has polygonal, oval or cuboidal cells and is associated with a better median overall survival of 13.1 months (1, 2). However, the sarcomatoid variant with spindle-shaped cells has a median survival of only 4 months (1). Both surgery and radiotherapy have limited roles in the management of the disease (3). VEGF inhibition in combination with chemotherapy results in a modest increase in survival for patients with malignant pleural mesothelioma (4). However, the first randomized trial of immune checkpoint inhibition using tremulimumab, an anti-CTLA-4 antibody, failed to improve median overall survival (5). In addition, nintedanib, a multi-tyrosine kinase small molecule inhibitor targeting VEGFR1-3, PDGFRα/β and FGFR1-3 receptor signaling, did not prolong progression-free survival when added to chemotherapy (6). Various Phase 2 trials, such as the MAPS2 trial of nivolumab and ipilimumab, show promising activity and require confirmation in larger Phase 3 trials (7), While Phase 1 and Phase 2 trials of immunotherapies have produced modest signals to date, checkpoint inhibition in real-life clinical settings have reported limited effects. For example, in Phase 1b and 2 trials of pembrolizumab, the median survival is between 11.5 and 18 (8, 9), but median survival is only 7.2 months when prescribed off-label in palliative settings (10). Furthermore, the results from the randomized Phase 3 PROMISE-meso trial indicated that pembrolizumab was not superior to single-agent chemotherapy in pre-treated MPM (11). While several trials using immunotherapy monotherapy, combination immunotherapy or immunotherapy in combination with chemotherapy are underway in mesothelioma, it is pertinent to examine the tumor immune microenvironment for explanations as to why mesothelioma is so resistant to therapy.

## The Inflammatory Response and Carcinogenesis

The inflammatory response to asbestos is a cardinal feature of mesothelioma's pathogenesis and microenvironment. The inflammatory response to asbestos fibers that reach the outer pulmonary parenchyma is one hypothesis for how amphibole fibers and fluid enter the pleural space in the first place (12). In addition, mesothelial cells in contact with asbestos fibers generate CCL2 (13), attracting macrophages which become embroiled in “frustrated phagocytosis” due to the size and biopersistence of amphibole fibers (12). Macrophage production of Reactive Oxygen Species (ROS) and nitrogen species augments the reactive oxygen/nitrogen species already catalyzed by the iron in asbestos fibers (14–18). The quantity of hydroxyl free radicals and nitric oxide free radicals have been associated with the extent of DNA strand breaks and gene deletions in cultured cell lines and are considered responsible for key mutagenic events (14, 15, 19).

Furthermore, cells which have sustained genotoxic damage would ordinarily undergo poly(ADP)ribose polymerase-induced programmed cell death (20) but are “rescued” by aspects of the inflammatory response. For example, macrophages are key producers of TNF-α (17), not only as a consequence of frustrated phagocytosis (21), but also in response to the release of High Mobility Group Box 1 from mesothelial cells undergoing programmed cell death (20). TNF-α acting on upregulated TNF-α receptors and the NF-κB pathway can protect human mesothelial cells from cell death *in vitro* (22). This effect can be abrogated by antibodies to TNF-α or inhibitors of NF-κB (22). While TNF-α receptor knockout mice have not yet been studied in mesothelioma models, these mice are protected from fibroproliferative lesions when exposed to asbestos (23). In summary, the innate immune system, particularly macrophages, contribute to a milieu that promotes mutagenesis as well as the survival of mutated mesothelial cells.

## Extracellular Matrix And Stroma—More Than a Scaffold

In mesothelioma, the surrounding stroma is not merely a scaffold but promotes tumor growth, invasion and protection from an anti-tumor immune response. Many genes related to the synthesis of, and interaction with, extracellular matrix (ECM) are upregulated in RNA expression analyses of mesothelioma specimens (24–27). These ECM-related genes are more associated with biphasic (25), desmoplastic (27) and sarcomatoid variants (27)—the histological subtypes with poorer prognoses. Mesothelioma cell lines can also produce various ECM components such as type IV collagen, laminin and fibronectin, as well as integrins which bind to these proteins (28, 29). ECM components have autocrine and paracrine effects that stimulate mesothelioma cell chemotaxis and haptotaxis (28, 29). Under the influence of various growth factors mesothelioma cell lines can also produce matrix metalloproteases (MMP) to remodel the ECM and permit invasion (30). Some of these MMPs such as MMP2 and MMP14 are also associated with a poorer prognosis in mesothelioma (31, 32). Furthermore, there is an association with these stroma-related genes and so-called “immune deserts,” tumor regions with little lymphocytic infiltrate, suggesting that the stroma and ECM are acting as a barrier to the immune response (26).

**Figure 1:**
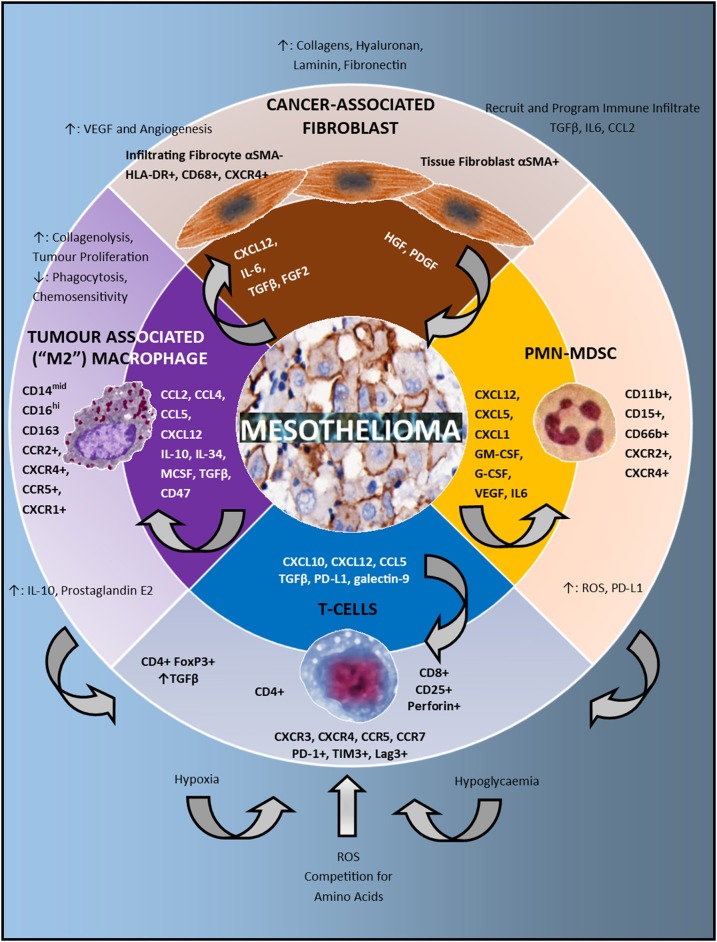
The immune microenvironment in mesothelioma. In the center of the schematic are mesothelioma cells. The second circle lists the chemokines, growth factors and checkpoints present in the microenvironment which attract and program the immune cell infiltrate. These cells include: cancer associated fibroblasts, Polymorphonuclear (PMN) Myeloid Derived Suppressor Cells (MDSC), T-cells and Tumor Associated Macrophages (TAMs). The direction of the arrowhead depicts which cells are influenced by these signals. The outermost circle describes both the phenotype and function of the immune infiltrate. Tumor associated macrophages have immunosuppressive effects on T-cells via increased IL-10 and prostaglandin E2 production. PMN-MDSC have immunosuppressive effects on T-cells via production of Reactive Oxygen Species (ROS) and upregulation of PD-L1. At the bottom of the schematic in blue, various metabolic factors also influence the activity of T-cells including hypoxia, hypoglycaemia, reactive oxygen species, and competition for amino acids.

When comparing mesothelioma tissue and cell lines, we can conclude that stromal cells and cancer-associated fibroblasts or fibrocytes contribute some of the signals seen in these RNA analyses (25). Activated fibroblasts are present in most mesothelioma tissues (33) and are identified by alpha smooth muscle actin (SMA). Although not studied in mesothelioma, two separate origins of cancer-associated fibroblasts and fibrocytes have been described: α-SMA expressing fibroblasts are tissue-derived, but fibrocytes with spindle-shaped nuclei are derived from macrophages or dendritic cells (α-SMA-, HLA-DR+ with moderate expression of CD68) (Figure 1) (34). Mouse models suggest that fibrocytes migrate to areas of hypoxia under the influence of CXCL12 and CXCR4 (35). Cancer-associated fibroblasts and fibrocytes can synthesize ECM components such as collagens, hyaluronan, laminin, and fibronectin and remodel ECM with MMP (36). Furthermore, these spindle-shaped stromal cells develop a positive-feedback relationship with tumor cells by secreting growth factors. For example, TGF-β and IL-6 are consistent features of the mesothelioma secretome (37) and are cardinal activating molecules for fibroblasts. In addition, Fibroblast Growth Factor 2 (FGF2) is seen in most mesothelioma tissue specimens by immunohistochemistry (IHC) (33, 38, 39) and leads to proliferation of fibroblast cell lines *in vitro* and migration to the malignancy in xenograft models in SCID mice (33). Furthermore, FGF2 leads to fibroblast production of hepatocyte growth factor (HGF) and platelet-derived growth factor A (PDGF-A) which can in turn stimulate the growth and migration of mesothelioma cell lines (33, 40). The HGF-receptor (c-MET) and the PDGF receptors α and β, are detected in the majority of mesothelioma specimens by IHC (41, 42). Unexpectedly, Phase 2 and Phase 3 clinical trials of PDGFR inhibition by the small molecular tyrosine kinase inhibitors vatalanib or nintedanib did not show major activity (6, 43). However, targeting FGFR using small molecules (44) or FGF-ligand “traps” (45), c-MET by tyrosine kinase inhibitors (46), or fibrosis with pirfenidone (47) continues to elicit considerable research interest.

Finally, in addition to molecules actively secreted by mesothelioma cells, cancer-associated fibroblasts have been noted to produce TGFβ, IL-6 and CCL2 (36). These molecules are detected in pleural effusions of patients with mesothelioma (37) and as such cancer-associated fibroblasts may contribute to the recruitment and differentiation of immunosuppressive cells. They can also contribute to VEGF production and subsequent angiogenesis (36, 37). In summary, the stroma and stromal cells provide a scaffold for invasion, a barrier to the immune response and stimulate tumor growth and the differentiation of immunosuppressive cells.

## The Mesothelioma Secretome And Metabolome

Before describing the cellular components of the tumor immune microenvironment, it is important to recognize that the chemotaxis and differentiation of these cells is influenced by chemokines, growth factors and metabolites. Examination of pleural fluid, patient-derived tumor cells and tumor cell lines are invaluable in evaluating the “secretome.” The mesothelioma secretome includes the chemokines CCL2, CCL4, CXCL10, CXCL5, CXCL1, and CXCL12, the cytokines IL-10 and IL-6, and the growth factors TGFβ, VEGF, MCSF, GM-CSF, G-CSF, FGF, and PDGF (33, 37, 48–53). These molecules can have autocrine effects and are responsible for the chemotaxis and differentiation of immune cells.

Hypoxia is one of the cardinal features of the mesothelioma metabolome. It is likely that tumor cells are exposed to fluctuating oxygen levels due to rapid tumor proliferation, stromal reactions, and angiogenesis (54). In patients with mesothelioma, this hypoxia is noted on F-fluoromisonidazole (FMISO) Positron Emission Tomography (PET) scans, and is associated with increased metabolic activity on Fluorodeoxyglucose (FDG)-PET (55). Evidence of hypoxia has also been demonstrated using immunohistochemical detection of Hypoxia Induced Factor 1α (HIF1α) (56). Hypoxia is capable of profoundly enhancing the growth of mesothelioma cell lines: including clonogenicity, stemness, resistance to chemotherapy, epithelial to mesenchymal transition, migration, morphological changes with pseudopodia, and various phenotypic changes (increased expression of HIF1α/2α, CD44 and Oct4, Bcl2, E-cadherin, vimentin and Glut1) (57). In addition, hypoxia results in the influx of additional immune cells via increased expression of CXCL12 (35) and stimulates angiogenesis by the upregulation of VEGF expression (54, 58). Furthermore, hypoxia, acting via increased HIF1α-expression, increases PD-L1 expression in tumor cell lines as well as in murine macrophage and dendritic cells (58). In myeloid derived suppressor cells (MDSCs), HIF1α expression is associated with increased *arg1* and *inos* and the suppression of T-cell proliferation in mice (59). Knockout of HIF1α was able to abrogate all these effects (59). Hypoxia also induces MDSC production of IL-6, IL-10, and TGFβ1 (58). Apart from MDSCs, murine macrophages exposed to hypoxia increase HIF1α expression and have enhanced suppression of T-cell proliferation (60). HIF1α knockout also abrogated this effect (60).

Apart from oxygen, infiltrating immune cells compete with mesothelioma cells for key nutrients. Mesothelioma cells can upregulate Glucose Transporter 1 (Glut1) in order to more efficiently access glucose and this is evident on IHC (61). Elevated Glut1 levels has been recognized as a poor prognostic factor (62). Mesothelioma is typically a low glucose environment and glucose is reduced in mesothelioma-associated pleural effusions (63). In such an environment, competition for glucose can substantially affect T-cell function (64). Similar competition occurs for essential amino acids. For example, mesothelioma can increase L-type Amino acid Transporter 1 (LAT1)-expression and this has also been associated with poor prognosis in univariate analyses (65). LAT1 transports both arginine and tryptophan and therefore the tumor can deprive T-cells of amino acids essential for T-cell proliferation and function (64). Mesothelioma cells may also express increased levels of Indoleamine-pyrrole 2,3-dioxygenase (IDO) (66) which metabolizes tryptophan into kynurenine, inhibiting T-cell glycolysis and function (64). To conclude, the mesothelioma secretome and metabolome both attract and program infiltrating immune cells.

## Immune Cell Infiltrate

### Tumor-Associated Macrophages

Tumor associated macrophages (TAMs) are prominent in the tumor microenvironment; they are associated with a poor prognosis and mouse models suggest that they could be a potential target for treatment. TAMs are generally the most prominent cells in the immune infiltrate when analyzed by flow cytometry of pleural effusions and constitute on average 26–42% of the cellular immune infiltrate in mesothelioma by IHC (51, 67–69). While not the subject of specific analysis in mesothelioma, most of the CD163+ TAMs in other malignancies are monocyte-derived from the peripheral blood rather that tissue-resident macrophages (34). Chemokine signals that attract monocytes in mesothelioma include CCL2, CCL4, CCL5, and CXCL12 and these appear to be of mesothelioma cell origin (Figure 1) (37, 52, 53). Murine experiments of asbestos-induced mesothelioma also implicate CCL7, CCL8, CCL3, and CX3CL1 but these have not been detected or investigated in humans to date (70). In relation to macrophages, CCL2 has been studied in most detail in mesothelioma with CCL2 concentrations in malignant pleural effusions being substantially higher compared to benign pleural effusions and pleural effusions from patients with lung adenocarcinoma (24, 71). CCL2 acting via CCR2 appears to be the key chemokine in monocyte trafficking in MPM. Monocytes migrate toward malignant pleural fluid or mesothelioma cell line supernatant and neutralizing antibodies to CCL2 or CCR2 substantially reduce this migration in Transwell experiments (48). However, CD14+ monocytes found in pleural and peritoneal effusions of patients with malignant mesothelioma are also noted to express CXCR4, CCR5, and CXCR1 with varying degrees of positivity in flow cytometry (72). Other chemokine receptors that can be found on monocytes, such as CX3CR1 and CCR1, are also upregulated in RNA-seq analyses of asbestos-induced mesothelioma in mice (70).

Monocytes and macrophages are programmed into suppressor cells by various components of the mesothelioma secretome (Figure 1). For example, primary cells from patients with MPM that are capable of producing M-CSF and IL-34, and MCSF can be detected in pleural effusions (48, 73). These growth factors are implicated in monocyte and macrophage development but may also have autocrine functions as well (73). Other key cytokines for macrophage activation such as TGF-β and IL-10 have been identified in pleural fluid and supernatant from mesothelioma cultures, also suggesting a tumor origin (51, 74). IHC of MPM samples have confirmed the presence of TGFβ (38) and this feature appears to distinguish MPM from primary lung cancers (74, 75). An autocrine feedback loop has also been proposed for TGF-β (76). Apart from the immunosuppressive and polarising cytokines described above, the macrophage checkpoint and “don't eat me signal,” CD47, was found to be expressed in high levels in the majority of patients with epithelioid mesothelioma (77).

TAMs develop an immunosuppressive phenotype in mesothelioma; human monocytes cultured with malignant pleural effusions developed a CD14^mid^CD16^hi^ immunosuppressive phenotype, resembling cells cultured with M-CSF (48). Furthermore, Izzi et al. performed a comprehensive array of macrophage function tests to show that co-culture of THP-1-derived macrophages with a single mesothelioma cell line resulted in reduced phagocytic activity, increased IL-10 production, increased collagenolytic activity for tissue remodeling, and increased arachidonic acid and prostaglandin E2 production (78). Curiously, contrasting effects were noted on monocytes (78). When co-cultured with immunosuppressive macrophages, mesothelioma cells proliferate more and have reduced sensitivity to chemotherapy with cisplatin or pemetrexed (48). The functional importance of macrophages in promoting mesothelioma is attested in a syngeneic, immunocompetent, orthotopic mouse model of mesothelioma (79). When the local macrophage population was selectively removed using liposome-encapsulated clodronate, reduced tumor number, invasiveness, and metastases were observed (79).

There have been conflicting reports on the prognostic effect of macrophages in epithelioid and non-epithelioid mesothelioma (68, 80). However, more precise biomarkers using an immunosuppressive to pan-macrophage ratio with CD163 to CD68 correlated with poor overall survival in a cohort of patients with epithelioid mesothelioma (81). Greater quantities of circulating monocytes are also associated with worse outcomes from cytoreductive surgery (68). The effect is associated with tumor bulk but is still seen when controlling for disease stage (68), suggesting that both tumor size and its distinct secretome could be influencing peripheral blood monocyte counts. A low peripheral blood lymphocyte-to-monocyte ratio has also been identified as a marker of poor prognosis (82). In summary, TAMs are numerous, programmed by the mesothelioma secretome, have an immunosuppressive phenotype and function, and are associated with poor prognosis.

### T-Lymphocytes

The CD3+ T-lymphocyte is the second most common immune cell present in the mesothelioma microenvironment and constitute on average 20–42% of the immune cell infiltrate (69, 80, 83). CD8+ T-cells are almost universally present and CD4+ and CD4+ FoxP3+ T-cells are also present in the majority of patients (67, 83). Of interest, the number of T-regulatory cells in pleural effusions of MPM patients is lower than in other solid tumors (74). With regards to T-cell trafficking, apart from CXCL12 discussed previously, the mesothelioma secretome also includes CXCL10 (37). CXCL10 is produced in greater concentrations in pleural fluid compared to the supernatant of primary cells, suggesting additional origins of the chemokine rather than solely from tumor cells (37). The CXCR3 chemokine receptor for CXCL10 is upregulated in murine models of asbestos-induced mesothelioma (70). CCL5 is also substantially elevated in the peripheral blood of patients with mesothelioma compared to asbestos workers and healthy individuals (84) and the CCR5 receptor is present on T-cells in pleural effusions (72). Other chemokine receptors on T-cells in pleural effusions include CXCR4 and CCR7 (72).

The mesothelioma microenvironment includes both neoantigenic stimuli as well as checkpoint molecules which can affect T-cell programming. Although next generation sequencing of mesothelioma originally identified few neoepitope generating mutations (85), more recently mate-pair seq based analysis has identified higher numbers of neoepitope generating mutations which were probably from chromosomal rearrangements missed by NGS (86). When analyzing predicted neoantigen load and TCRβ diversity in MPM, it is noted that in general the most diverse polyclonal TCRβ repertoire is associated with fewer predicted neoantigens. In contrast oligoclonal expansion is associated with high neoantigen loads presumably due to clonal expansion (87). While neoantigens may prompt T-cell activation and proliferation, various checkpoint molecules are also evident in the mesothelioma microenvironment and are discussed in more detail elsewhere in this issue. PD-L1 is detected by flow cytometry of pleural effusions as well as IHC (88–91) and has been associated with poor prognosis (88, 89). Of interest, PD-L1 expression is associated with a higher objective response rate to nivolumab but is not entirely predictive of response (7). This finding is reflected in other malignancies treated with PD-1 or PD-L1 inhibition, indicating that other parameters including tumor mutational burden or tumor-infiltrating lymphocytes also influence response to PD-1 or PD-L1 blockade (92, 93). Galectin 9, a ligand for TIM-3 has also been detected by IHC and by flow cytometry on human macrophages (94). T-regulatory cells are consistently detected in MPM IHC and flow cytometry of associated pleural effusions (37, 67, 74, 80). The T-regulatory compartment develops in the context of abundant TGF-β and presumed inadequate stimulation by dendritic cells (37, 74). It has also been shown that PD-L1 signaling via PD-1 is responsible for the plasticity of some TH1 cells, converting them to inducible T-regulatory cells (95).

As a result of the above influences, the phenotype of infiltrating T-cells is varied. The CD8+ T-cells that are present in pleural effusions show higher levels of CD25+ compared to other malignancies, generally indicative of activation (74). In addition, there is an increase in perforin expression in CD8+ T-cells which correlated with the number of neoepitopes that are present in the tissue (87). Despite these signs of activation, CD8+ cytotoxic T-cells also display phenotypic markers of exhaustion including PD-1+, TIM3+, and LAG3+ (88). CD4+ T-helper subsets and function in mesotheliomas have not been extensively investigated but again clear signs of exhaustion are evident with significant levels of PD-1+, TIM3+, and LAG3+ detected by flow cytometry (88). Of the T-cells present in mesothelioma, the majority have an effector memory phenotype (69).

Although one cannot draw conclusions regarding causation, T-cell numbers are associated with patient prognosis. Two studies have shown that epithelioid mesotheliomas infiltrated by more CD4+ T-cells were associated with a better prognosis (67, 80). A third study showed an association with prognosis that was only statistically significant in univariate analysis (53). This association has not been confirmed in sarcomatoid tumors (80). Only one comparatively small study demonstrated a poorer prognosis in multivariate analyses of low CD8+ T-cell counts (83). Interestingly, low CD8+ T-cell count was also a poor prognostic factor in patients undergoing extrapleural pneumonectomy (96). High proportions of FoxP3 positive T-cells have been associated with a poor prognosis in analyses of epithelioid and sarcomatoid tumors (80).

Although it is presumed that this T-cells infiltrate has some functional significance, the clinical experience with intrapleural IL-2 has been disappointing. While there is yet to be any randomized trial of IL-2, in one study the overall survival did not differ substantially from historical controls who underwent the same intensive therapy with pleural decortication, intrapleural postoperative epidoxorubicin, adjuvant radiotherapy followed by chemotherapy and did not receive any IL-2 (97). Immunological effects seen in response to IL-2 include an increase in both CD8+ T-cells as well as FoxP3+ T-cells (97). This suggests that the T-regulatory cells are acting as a “sump” for IL-2 in this context. There is also conflicting evidence regarding the effects of anti-CD25 therapy in murine experiments (98, 99). In summary, T-lymphocytes are programmed by the mesothelioma secretome, neoantigens and checkpoint molecules and are associated with altered prognosis. The remaining challenge is to determine whether they can be successfully redirected into a robust anti-tumor response.

Chimeric Antigen Receptor (CAR) T-cell therapy is one such method of enhancing patient T-cell responses against mesothelioma and is discussed in more detail elsewhere in this issue. The requirement for neoantigens is bypassed by directing the CAR T-cell receptor to a tumor-associated antigen, such as mesothelin. The fibrous stroma can be circumvented by locoregional administration (100, 101), or designing CAR T-cells to target antigens that are expressed by both the tumor and cancer-associated stroma such as Fibroblast Activation Protein (102), or by adding chemokine receptors such as CCR2 to enhance trafficking to tumor (103). T-cell metabolism can be manipulated by the choice of costimulatory molecules, such as 4-1BB (104, 105). Exhaustion can also be ameliorated by the concomitant use of PD-1 inhibitors (100, 101), or designing CAR T-cells with dominant negative PD-1 receptors to prevent signaling via native PD-1 (100). Switch receptors have also been designed for mesothelin CAR T-cells with extracellular PD-1 linked to intracellular CD28 (106). Other modifications such as mutating the CAR CD3ζ Immunoreceptor Tyrosine-Based Activation Motifs have also been shown to prevent exhaustion in other disease models (107), and these principles are likely to be applicable to mesothelioma. These developments address some challenges posed by the tumor microenvironment and results of early clinical trials are eagerly anticipated.

### Myeloid-Derived Suppressor Cells

Myeloid-derived suppressor cells (MDSC) can be polymorphonuclear (PMN-MDSC) or monocytic (M-MDSC). However, the distinction between MDSC and other immune cells such as TAMS is still unclear despite proposed standardized nomenclature and markers for identification (108). The granulocytic infiltrate is less prominent and on average is 6–9% of the cellular infiltrate (49, 69) but still has prognostic implications and functional importance. Neutrophilic infiltrate can be detected by IHC, perhaps with greater sensitivity using CD66b (which also detects eosinophils) and CD15 compared to neutrophil elastase (49, 69, 80). Apart from CXCL12 and CXCR4 previously mentioned, other neutrophil chemoattractants include CXCL5 and CXCL1 which are detected in patient-derived mesothelial cell supernatants, and CXCL5 also reaches detectable levels in pleural effusion (37). Murine mesothelioma models show upregulation of the granulocyte chemokine receptor CXCR2 for these ligands (70).

Granulocytic growth factors are produced in the mesothelioma secretome including GM-CSF, G-CSF, VEGF, and IL-6 (37, 49). Furthermore, in the mesothelioma microenvironment granulocytes develop a phenotype consistent with PMN-MDSC and express CD15+, CD11b+, CD66b+, and are CD14/CD33 double-negative (49, 108). These polarizing growth factors likely have systemic effects as increased populations CD11b+CD15+HLADR- granulocytes are also noted in the peripheral blood of patients with mesothelioma compared to healthy controls (49). These cells function as MDSCs and inhibit the proliferation of T-cells compared to CD15+ cells from normal pleura or from the peripheral blood of healthy donors (49). The inhibitory effect of these MDSC is predominantly through the generation of ROS; peripheral blood granulocytes from patients with MPM show increased ROS expression and the proliferation of T-cells can be restored with inhibitors of ROS such as N-Actyl Cysteine (49). Free radical species can also affect T-cell function by nitration of the T-cell receptor (109), downregulation of CD3ζ, and H_2_O_2_-mediated reduction in cytokine production (110). PD-L1 expression on granulocytes has also been associated with fewer T-cells in the tumor (49). While various alternative mechanisms of immunosuppression have been attributed to MDSCs, *in vitro* assays with peripheral blood granulocytes indicate that immunosuppressive cytokines, arginase expression or iNOS expression were the same in patients and healthy controls (49). Moreover, arginase or iNOS inhibitors did not restore T-cell function (49). However, it is important to note is that these experiments assessed peripheral blood granulocytes in patients rather than tumor-associated MDSCs. The presence of greater neutrophilic infiltrate in tumor and an increased peripheral blood neutrophil to lymphocyte ratio is associated with a poorer prognosis in epithelioid mesothelioma (80, 111).

Chemotherapies that are recognized to reduce MDSCs have been used to treat MPM. 5-Fluorouracil or paclitaxel did not show positive effects whereas mixed results were seen with gemcitabine (112). In summary, PMN-MDSC are relatively abundant and are also associated with prognosis. However, it is remains to be seen if eliminating these cells with targeted therapy will be successful.

### Other Cells

B-cells have been detected in both tumor and stroma in MPM to varying degrees (26, 53, 69, 80). Higher B-cell counts have been associated with a better prognosis in multivariate analyses of patients with epithelioid mesothelioma (53, 80). However, it is yet to be determined whether this is an epiphenomenon or whether the B-cells themselves have a functional role. Autoantibodies have been detected in the sera of a fraction of patients with mesothelioma (113). Some of these antibodies appear to be tumor-specific and target the nuclear fraction (113). However, in a more comprehensive analysis of sera from patients with MPM against a limited panel of autoantigens, the percentage of patients with autoantibodies was not markedly elevated compared to other patients with asbestos-related diseases or asbestos-exposed healthy controls (114). The antibody subclasses from B-cells taken from mesothelioma tissues appear to be predominantly IgG1 and IgG3 which are known to activate complement (115). The analysis of B-cell cytokines or B-regulatory cells is currently limited in mesothelioma (116).

CD3-CD56+ Natural Killer (NK) and CD3+CD56+ Natural Killer T (NKT) cells are found in the majority of mesothelioma tissues but only in very small numbers (69, 80, 96, 117, 118). In pleural effusions they are found to have typical inhibitory receptors (NKG2A) and activation receptors (NKG2D) but are also CD56^bright^, a subset associated with poorer cytotoxicity but enhanced cytokine production (117). A greater proportion of peripheral blood NK cells also express the exhaustion marker TIM3+ (119). While pleural effusion NK cell function is reduced in degranulation assays compared to the peripheral NK cells from healthy donors, similar changes were noted in NK cells from non-malignant pleural effusions (117). The interpretation of these data is problematic given that there is no healthy control or reference range for pleural NK cell cytotoxicity (117). However, it is noteworthy that after treatment with IL-2 *in vitro*, the cytotoxicity of NK cells from various malignant effusions can be restored, suggesting some reversibility in impaired function (120). In murine mesothelioma tumor models, removing NK cells by anti-asialo GM1 antibodies did not alter tumor growth, nor was tumor growth accelerated in beige mice with impaired NK cell function (121). The presence of NK cells as detected by IHC has also not been associated with altered prognosis in either epithelioid or sarcomatoid mesothelioma (80). In conclusion, current evidence does not indicate that NK cells are key players in the mesothelioma tumor microenvironment.

Mast cells have been detected in mesothelioma tumors treated with IL-2 and high counts of tryptase-positive mast cells has been associated with a better prognosis but this is awaiting further confirmation (122). Dendritic cells do not constitute a large population in the mesothelioma tumor microenvironment when assessed with antibodies to CD123 in IHC (69).

While this review focuses on the immune aspects of tumor microenvironment, it is prudent to acknowledge that angiogenesis is a simultaneous and interlinked process that also requires therapeutic intervention. In fact, immunosuppression and angiogenesis are intrinsically interconnected repair mechanisms co-opted by malignancy (123). Both have linked physiological roles, but both occur in an unchecked and disorganized manner in the context of the tumor microenvironment (123). As we have discussed, both share metabolic and growth factor stimuli, such as hypoxia, VEGF, HGF, TGF-β, angiopoietin, and prostaglandin E2 (37, 123–125). Studies in mesothelioma and other malignancies indicate that both processes are driven by tumor cells, cancer associated fibroblasts, MDSCs, TAMS, and T-regulatory cells (33, 36, 126, 127). In addition, angiogenesis measured by microvessel density is an independent marker of poor prognosis in mesothelioma (128) and anti-angiogenic therapy with Bevacizumab improves median overall survival (4). While anti-angiogenic therapies in mesothelioma require further refinement and are discussed elsewhere in this edition, it is likely that successful immune-based treatments would also benefit from incorporating ancillary anti-angiogenic treatments.

## Conclusions

While checkpoint inhibition represents an exciting development in the treatment of several solid tumors, the outcomes in mesothelioma have been less positive and may well be affected by the complex structure of the tumor microenvironment in mesothelioma. While more comprehensive descriptions of the tumor microenvironment and suppressor cells have been presented elsewhere, we have chosen to focus on research that relates specifically to mesothelioma, given the evidence that MPM poses unique challenges when compared to other malignancies. We recognize that this review may not adequately emphasize the significant heterogeneity between patients and within the tumor microenvironment itself. However, we hope that providing a better understanding of the stromal tissue, the secretome, metabolome and relevant immunosuppressive cells will assist in finding the rationale for more effective therapy combinations in the future.

## Author Contributions

GC wrote the first draft of the manuscript. All authors contributed to manuscript revision, read, and approved the submitted version.

### Conflict of Interest

The authors declare that the research was conducted in the absence of any commercial or financial relationships that could be construed as a potential conflict of interest.

## Abbreviations

CTLA-4: Cytotoxic T lymphocyte Associated Protein
ECM: Extracellular Matrix
FGF: Fibroblast Growth Factor
G-CSF: Granulocyte Colony Stimulating Factor
GM-CSF: Granulocyte and Macrophage Colony Stimulating Factor
HGF: Hepatocyte Growth Factor
iNOS: Inducible Nitric Oxide Synthase
M-CSF: Macrophage Colony Stimulating Factor
MMP: Matrix Metalloproteases
MPM: Malignant Pleural Mesothelioma
NF-κB: Nuclear Factor Kappa-Light-Chain-Enhancer of Activated B cells
PD-1: Programmed Cell Death Protein 1
PD-L1: Programmed Death Ligand 1
PDGF: Platelet Derived Growth Factor
PMN-MDSC: Polymorphonuclear Myeloid Derived Suppressor Cells
ROS: Reactive Oxygen species
SMA: Smooth Muscle Actin
TAM: Tumor Associated Macrophages
TIM3: T-cell Immunoglobulin and Mucin-Domain Containing-3
TGFβ: Transforming Growth Factor β
VEGF: Vascular Endothelial Growth Factor.
